# Saponins Extracted from Tea (*Camellia Sinensis*) Flowers Induces Autophagy in Ovarian Cancer Cells

**DOI:** 10.3390/molecules25225254

**Published:** 2020-11-11

**Authors:** Yaomin Wang, Chen Xia, Lianfu Chen, Yi Charlie Chen, Youying Tu

**Affiliations:** 1 Department of Tea Science, Zhejiang University, Hangzhou 310058, Zhejiang, China; wangym@mail.hzau.edu.cn (Y.W.); lukexia@tealab.cn (C.X.); c.lianfu@foxmail.com (L.C.); 2 Key Laboratory of Horticulture Plant Biology, Ministry of Education, College of Horticulture & Forestry Sciences, Huazhong Agricultural University, Wuhan 430070, Hubei, China; 3 Key Laboratory of Urban Agriculture in Central China, Ministry of Agriculture, Wuhan 430070, Hubei, China; 4 College of Health, Science, Technology and Mathematics, Alderson Broaddus University, Philippi, WV 26416, USA

**Keywords:** saponins, phytochemicals, tea (*Camellia sinensis*) flower, ovarian cancer, autophagy

## Abstract

Tea flower saponins (TFS) possess effective anticancer properties. The diversity and complexity of TFS increases the difficulty of their extraction and purification from tea flowers. Here, multiple methods including solvent extraction, microporous resin separation and preparative HPLC separation were used to obtain TFS with a yield of 0.34%. Furthermore, we revealed that TFS induced autophagy—as evidenced by an increase in MDC-positive cell populations and mCherry-LC3B-labeled autolysosomes and an upregulation of LC3II protein levels. 3-MA reversed the decrease in cell viability induced by TFS, showing that TFS induced autophagic cell death. TFS-induced autophagy was not dependent on the Akt/mTOR/p70S6K signaling pathway. TFS-induced autophagy in OVCAR-3 cells was accompanied by ERK pathway activation and reactive oxygen species (ROS) generation. This paper is the first report of TFS-mediated autophagy of ovarian cancer cells. These results provide new insights for future studies of the anti-cancer effects of TFS.

## 1. Introduction

Epithelial ovarian cancer continues to be a deadly disease due to its high mortality rate and poor long-term prognosis [1,2,3,4]. In recent years, much attention has been focused on studying natural products and searching for compounds which possess anti-cancer effects [5,6,7]. The effectiveness of tea (*Camellia sinensis*) against different types of cancer has been extensively studied owing to the numerous bioactive constituents contained in the tea plant [8,9,10,11]. Tea flowers are the reproductive organs of tea, and recent studies have demonstrated the effective anti-cancer effects of tea flowers and compounds extracted from tea flowers [12]. Tea flowers contain a number of bioactive constituents including polyphenols, polysaccharides, amino acids and saponins [13,14,15,16]. Tea flower saponins in particular have attracted attention for their anticancer effects [17]. However, the diversity and complexity of tea flower saponins increases the difficulty of their extraction and separation from tea flowers, which limits the research of tea flower saponins.

Apoptosis and autophagy are two major modalities of programed cell death (PCD). Targeting PCD pathways is becoming an important strategy for drug discovery from natural compounds [18]. Apoptosis and autophagy may share common upstream signals; thus, apoptosis and autophagy may occur simultaneously [19,20,21]. Natural saponins have been reported to induce combined apoptosis and autophagy in various cancer cell lines in vitro [22,23,24]. Our previous study revealed that tea flower saponins (TFS) induced apoptosis in A2780/CP70 human ovarian cancer cells and OVCAR-3 cells, as well as S-phase arrest [17]. Nonetheless, an autophagic effect caused by TFS has never been reported.

In this study, we extracted and isolated TFS from tea flowers, then demonstrated for the first time that TFS induced autophagy, and evaluated the molecular mechanisms in OVCAR-3 human ovarian cancer cells.

## 2. Results

### 2.1. Extraction of TFS from Tea Flowers

**Figure 1:**
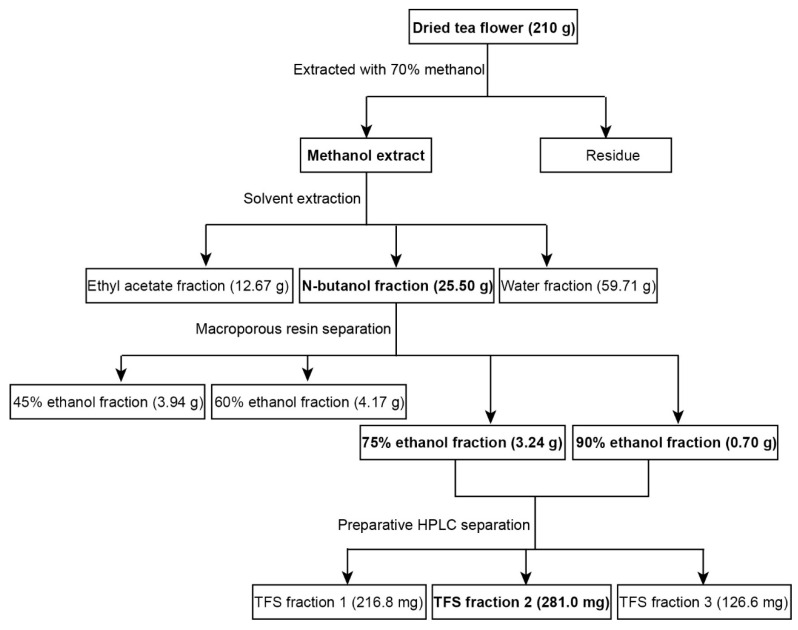
Extraction and isolation scheme of tea flower saponins (TFS) from tea flowers.

Multiple processes were used to extract highly purified TFS from tea flowers. As shown in Figure 1, 210 g dried tea flowers were used for the extraction of TFS, which involved 70% methanol extraction followed by solvent extraction and then microporous resin separation. TFS was mainly present in the 75% and 90% ethanol fractions. The 45% and 60% ethanol fractions also contained smaller amounts of TFS (Figure S3). We thus collected the 75% and 90% ethanol fractions for subsequent HPLC separation, resulting in three TFS fractions. We previously characterized the high purity TFS fraction 2 (Figure S2) and found it contains 14 triterpenoid saponins [17]. TFS fractions 1 and 3 also have high purities (Figure S4). The total yield was 0.34%. 

### 2.2. TFS Decreased Cell Viability and Induced Morphological Changes in OVCAR-3 Cells

**Figure 2:**
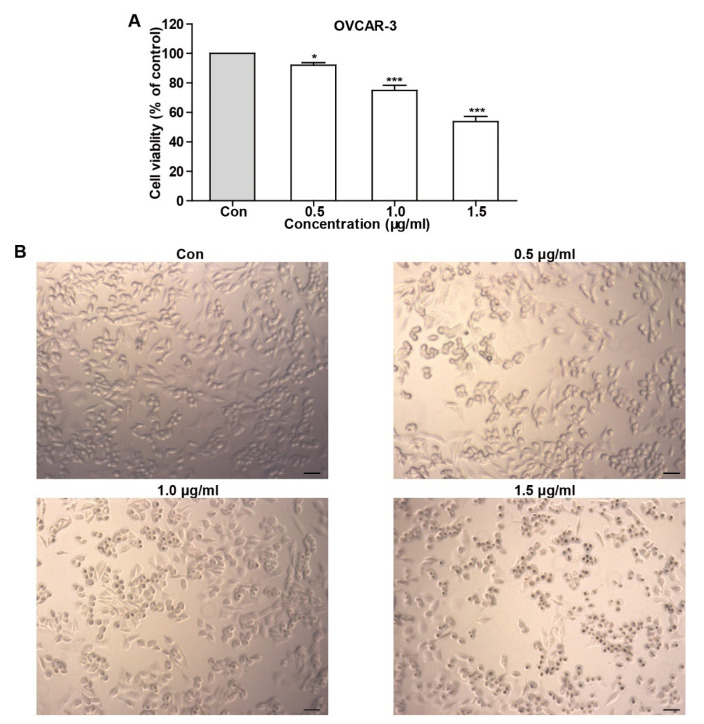
TFS decreased cell viability and induced morphology changes in OVCAR-3 cells. (**A**) The effect of TFS on cell viability was conducted using an MTS assay. Results were obtained from three independent experiments and expressed as mean ± SEM. Significant difference verse control (* *p* < 0.05 and *** *p* < 0.001. (**B**) OVCAR-3 cells were treated with the indicated concentrations of TFS for 24 h. Cell morphology was observed using microscopy (magnification 10×), scale bar = 50 μm.

To test whether TFS possesses an anti-cancer effect, we determined cell viability using an MTS assay after OVCAR-3 cells were treated with TFS for 24 h. As shown in Figure 2, TFS decreased cell viability in a dose-dependent manner. We additionally observed the morphology of OVCAR-3 cells by microscopy after treatment with TFS for 24 h. As shown in Figure 2, TFS induced morphological changes in OVCAR-3 cells, as the cells exhibited round bodies and clustered in groups.

### 2.3. TFS Triggers Autophagy in OVCAR-3 Cells

**Figure 3:**
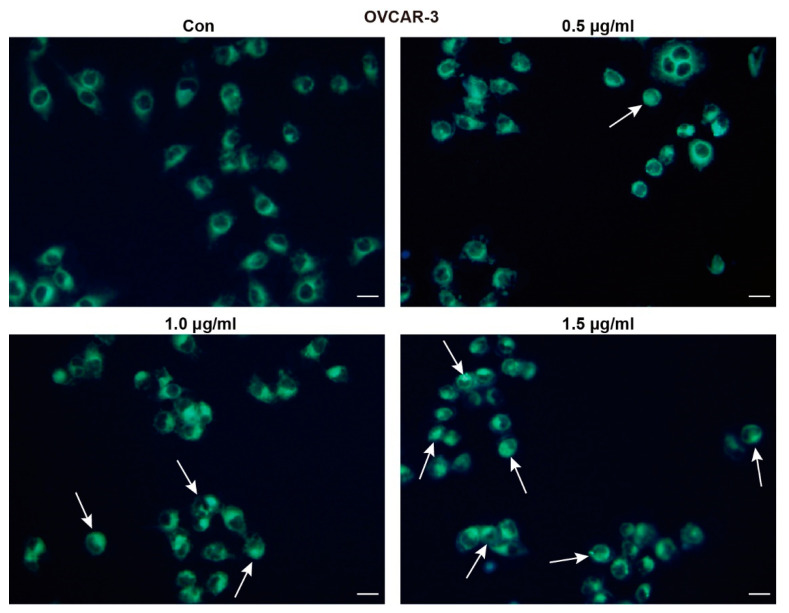
TFS induced autophagic vacuoles in OVCAR-3 cells. OVCAR-3 cells were treated with TFS (0, 0.5, 1.0 and 1.5 μg/mL) for 24 h then stained with MDC dye and visualized by fluorescence microscopy. White arrows indicate autophagic cells (magnification 20×), scale bar = 20 μm.

MDC staining was conducted to determine whether TFS induces autophagy and decreased viability of OVCAR-3 cells. MDC is an eosinophilic fluorescent dye that binds to the ubiquitin-like binding system Apg8, which requires the formation of autophagic vacuoles. Under excitation by ultraviolet light, the autophagic vacuoles demonstrated bright green fluorescence [25]. As shown in Figure 3, the presence of MDC-positive cells increased dose-dependently following treatment with TFS for 24 h. As indicated by the white arrows, bright green fluorescence appeared within the cells. The number of autophagic cells in the group treated with 1.5 μg/mL TFS for 24 h was significantly greater than the number of autophagic cells in the control group.

**Figure 4:**
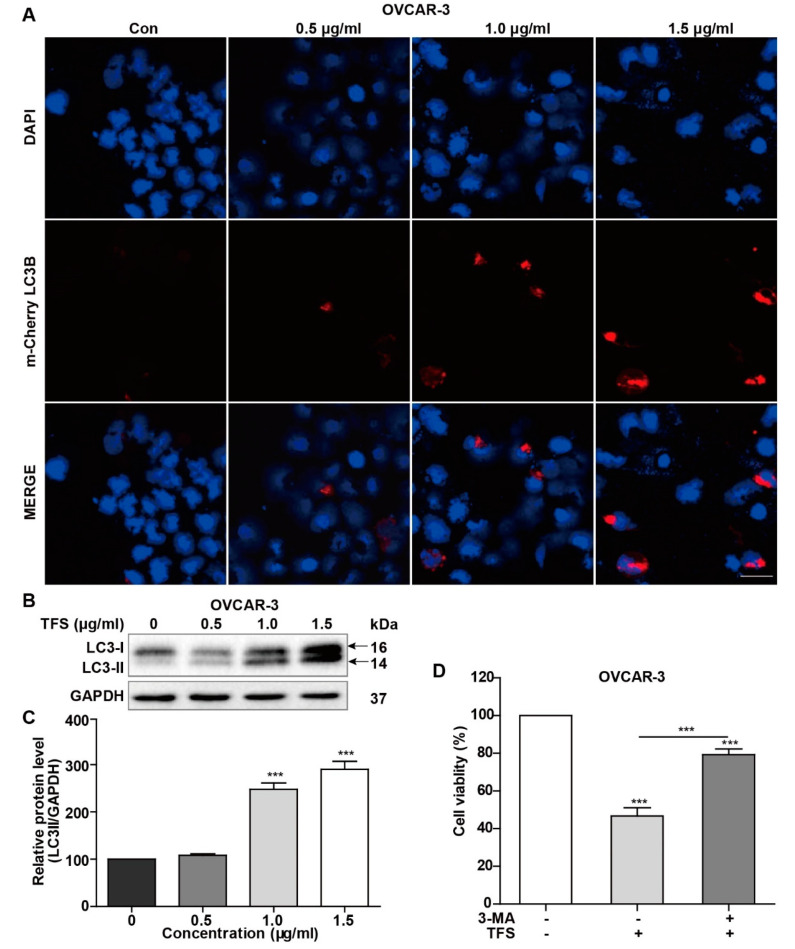
TFS treatment induced autophagic cell death in OVCAR-3 cells. (**A**) OVCAR-3 cells were transfected with mCherry-LC3B plasmid and treated with TFS (0, 0.5, 1.0, 1.5 μg/mL) for 24 h. Cells were imaged with an inverted laser scanning confocal microscope (magnification 40×), scale bar = 50 μm. (**B**,**C**) OVCAR-3 Cells were treated with different concentrations of TFS (0, 0.5, 1.0, 1.5 μg/mL) for 24 h. Western blotting was conducted to evaluate the levels of LC3I and LC3II protein with GAPDH used as an internal control. (**D**) OVCAR-3 cells were treated with 3-MA and TFS (1.5 μg/mL) for 24 h and then detected for cell viability. Each data point represents three independent experiments, significant difference versus control (*** *p* < 0.001).

To further confirm the induction of autophagy by TFS, autophagic lysosomes were detected by mCherry-LC3B plasmid transfection. The mCherry-LC3B fusion fluorescent protein aggregates on autophagic lysosomes during autophagy, and specific spots of bright red fluorescence can be visualized under confocal microscopy. In this study, mCherry-LC3B plasmids were transfected into OVCAR-3 cells, and then the cells were treated with TFS for 24 h. Confocal microscopy images were captured with an inverted laser scanning confocal microscope. As shown in Figure 4A, the number of autophagic lysosomes marked by red fluorescence in the OVCAR-3 cells increased with higher concentrations of TFS. 

We quantified the effect of TFS treatment on the expression of LC3 protein in OVCAR-3 cells by western blotting. Under normal conditions, LC3 protein is cleaved by Atg4 at the carboxyl end to form LC3-I (16 kD) and localizes to the cytoplasm. When autophagy occurs, ubiquitin-like systems such as Atg7 and Atg3 modify LC3-I, causing it to bind with phosphatidylethanolamine found on the surface of the autophagosome membrane to produce LC3II (14 kD) where it remains localized on the autophagic membrane. LC3-II expression is directly proportional to the activity of autophagic vesicles. Therefore, the change of expression between LC3-I and LC3-II could indicate whether autophagy is occurring. As shown in Figure 4B,C, the expression of LC3-II protein in OVCAR-3 cells increased significantly after treatment with TFS in a dose-dependent manner after 24 h. Taken together, the results from MDC staining, mCherry-LC3B plasmid transfection, and Western blotting for LC3 protein clearly indicated that TFS induced autophagy in OVCAR-3 cells.

Next, we added the autophagy inhibitor 3-MA to determine whether TFS-induced autophagy in OVCAR-3 cells promoted cancer cell survival or caused cancer cell death. As shown in Figure 4D, cell viability increased from 47% to 79% following treatment with 1.5 μg/mL of 3-MA for 24 h, indicating that TFS-induced autophagy promoted cell death of OVCAR-3 cells. Thus, these results revealed that TFS treatment led to the autophagic death of OVCAR-3 cells.

### 2.4. TFS-Induced Autophagy in OVCAR-3 Cells Independently from the Akt/mTOR/p70S6K Pathway and Was Accompanied by ERK Activation

**Figure 5:**
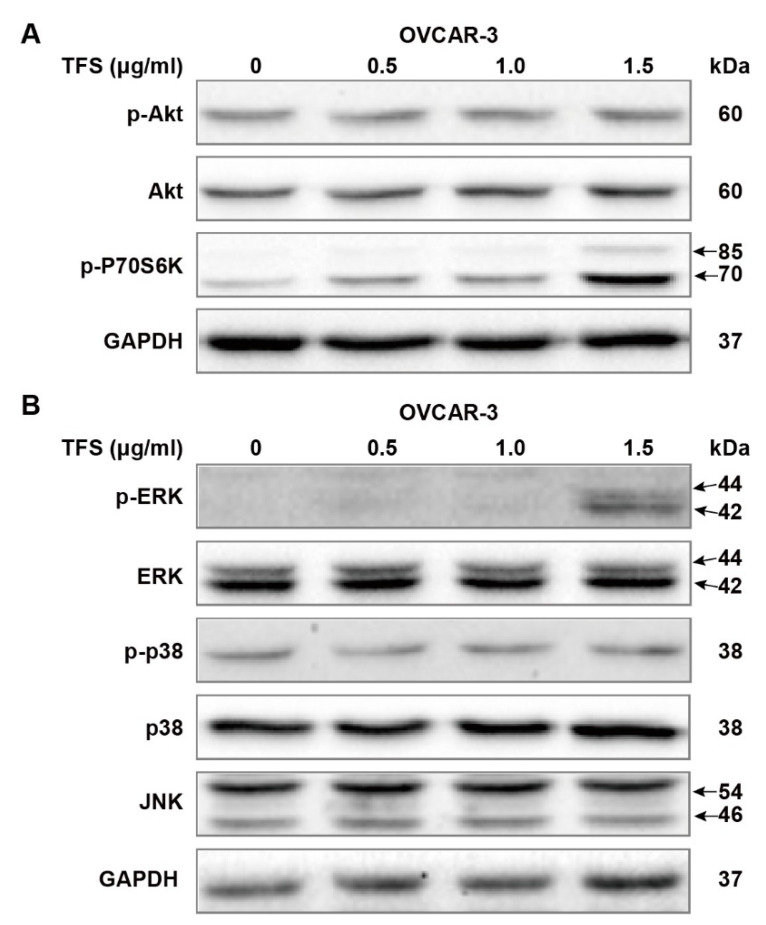
Effects of TFS on Akt pathways and MAPK pathways in OVCAR-3 cells. Protein expression of (**A**) Akt pathways related proteins p-Akt, Akt, p-P70S6K and (**B**) MAPK pathways related proteins p-ERK, ERK, p-p38, p38, JNK were analyzed by Western blot after being treated with different concentrations of TFS (0, 0.5, 1.0, 1.5 μg/mL) for 24 h, GAPDH was used as internal control.

We further evaluated the possible molecular mechanisms of TFS-induced autophagy by Western blot analysis, first by measuring Akt/mTOR/p70S6K pathway-related proteins. The Akt/mTOR/p70S6K pathway is a major autophagy regulatory pathway which negatively regulates autophagy [24,26,27]. As shown in Figure 5A, protein levels of p-Akt and Akt remained unchanged following treatment with TFS (0, 0.5, 1.0, 1.5 μg/mL) for 24 h. Phosphorylation of p70S6K was upregulated dose-dependently by TFS in OVCAR-3 cancer cell lines. Taken together, these results demonstrated that TFS-induced autophagy in OVCAR-3 cells was independent of the Akt/mTOR/p70S6K pathway. It has been reported that MAPK signaling pathways, which include ERK1/2, p38, and JNK, play a pivotal role in autophagy induction [22,27,28]. Accordingly, we evaluated the levels of MAPK signaling pathway proteins following treatment with TFS for 24 h. As shown in Figure 5B, TFS treatment significantly upregulated protein levels of p-ERK while total ERK expression remained unchanged. No obvious changes to p-p38, p38, and JNK expression were observed. These results indicated that the ERK pathway was activated in OVCAR-3 cancer cells. 

### 2.5. TFS-Induced Autophagy in OVCAR-3 Cells Is Accompanied by ROS Generation

**Figure 6:**
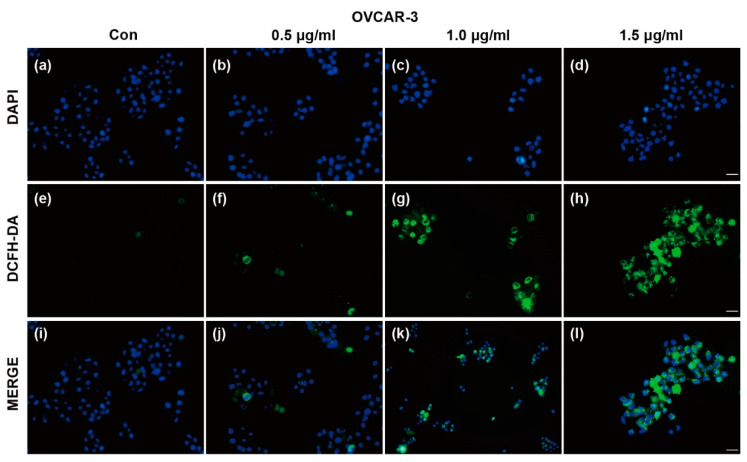
Effects of TFS on reactive oxygen species (ROS) generation in OVCAR-3 cells. ROS generation in OVCAR-3 cells was evaluated by DAPI and DCFH-DA double staining. Representative images are shown. After treatment with TFS (0, 0.5, 1.0 and 1.5 μg/mL) for 24 h, cells were stained with DCFH-DA and DAPI and visualized by fluorescence microscopy (magnification 20×), scale bar = 50 μm. The top line of images (**a**–**d**) shows cells stained with DAPI. The middle line of images (**e**–**h**) shows cells which generated ROS as represented by staining with DCFH-DA. The bottom line of images (**i**–**l**) shows merged fluorescence images of cells stained by DAPI and DCFH-DA.

Reactive oxygen species (ROS) generation is considered to be associated with the autophagy initiation and contributes to autophagy-mediated cell death [5,29]. We measured ROS generation using DCFH-DA following treatment with TFS. As shown in Figure 6, DCFH-DA positive cells increased after treatment with TFS, indicating that TFS treatment significantly enhanced ROS generation in OVCAR-3 cells. The results suggested that ROS generation is possibly involved in TFS-induced autophagy and this finding will need to be further elucidated. 

## 3. Discussion

Natural saponins from tea (*Camellia sinensis*) flowers possess remarkable structural diversity and have become increasingly important compounds for the prevention and treatment of cancer and other diseases [29,30,31]. Several triterpenoid saponins have demonstrated the ability to induce autophagy of cancer cells in various cancer cells lines [32,33]. To better understand the mechanisms underlying this observed anticancer effect, we performed a chemical extraction of triterpenoid saponins from tea flowers. We first extracted dried tea flowers with 70% methanol, which was the most efficient extraction solution according to our previous studies. We then performed solvent extraction, microporous resin separation, and preparative HPLC separation to obtain three TFS fractions with a total yield of 0.34%. Our previous study showed that TFS induced apoptosis and S phase arrest in A2780/CP70 and OVCAR-3 cells [17]. In our present study, we have shown for the first time that TFS can induce autophagic cell death in OVCAR-3 cells.

To confirm that TFS induced autophagy in OVCAR-3 cells, MDC staining was conducted following treatment with TFS for 24 h, showing a dramatic increase of MDC-positive cells. Autophagic lysosomes in OVCAR-3 cells increased significantly after transfection with mCherry-LC3B plasmids and TFS treatment. Western blot analysis was conducted to evaluate expression changes of LC3 in OVCAR-3 cells. LC3-II is considered to be the most reliable autophagic biomarker as LC3-II expression levels are proportional to the formation of autophagosomes [18,33]. In our study, we found that TFS dose-dependently upregulated protein levels of LC3-II in OVCAR-3 cells. These results indicated that TFS induced autophagy in ovarian cancer cells. Moreover, the autophagy inhibitor 3-MA reversed the decrease of cell viability caused by TFS, which further indicated that TFS induced autophagic cell death in OVCAR-3 cells.

Inhibition of the Akt/mTOR/p70S6K pathway is crucial for the induction of autophagy. p70S6K is the downstream target of Akt, and expression levels of p-p70S6K can be used as a marker for mTOR activity [22]. The natural compound delicaflavone can induce autophagy via the Akt/mTOR/p70S6K pathway in human lung cancer cells [26]. However, in our study, proteins levels of p-Akt and Akt remained unchanged while p-P70S6K was upregulated in OVCAR-3 cells. These results suggest that TFS-induced autophagy in ovarian cancer cells does not involve the Akt/mTOR/p70S6K pathway, which is consistent with previous reports that Platycodin D triggered autophagy in HepG2 cells [34]. 

We further evaluated the effects of TFS on MAPK signaling pathways and ROS generation. ERK, p38, and JNK are important regulatory proteins of MAPK signaling pathways which could also regulate autophagy processes [35,36]. In our present study, TFS significantly upregulated protein levels of p-ERK in OVCAR-3 cells. Protein levels of p-p38, p38, and JNK were not affected after treatment with TFS. These results indicated that the ERK pathway was activated by TFS-induced autophagy in ovarian cancer cells. Our findings were also consistent with several reports that ERK activation occurs in autophagy induced by different compounds in various cancer cell lines [22,37,38]. To better elucidate the role of ERK pathway in TFS-induced autophagy, ERK inhibitor such as U0126 should be applied to future experiment and clarify whether ERK inhibition attenuates TFS-induced autophagy in ovarian cancer cells. Additionally, TFS treatment significantly enhanced ROS generation in OVCAR-3 cells. As ROS generation is considered to be an essential aspect of apoptosis and autophagy [29], we previously reported that TFS induces apoptosis in A2780/CP70 and OVCAR-3 cells [17]. The specific role of ROS generation in TFS-induced cell death however, needs to be further elucidated by pretreatment with ROS inhibitor N-acetyl-L-cysteine (NAC), then to detect the expression levels of autophagy related proteins to clarify the role of ROS in TFS-induced autophagy in OVCAR-3 cells. Taken together, we conclude that TFS-induced autophagy in OVCAR-3 cells is accompanied by ERK activation and ROS generation, the mechanisms of which will merit further study.

## 4. Materials and Methods 

### 4.1. Materials and Reagents

Dried tea flowers (Figure S1) were purchased from Zhejiang Yilongfang Co., Ltd. (Hangzhou, China). Analytical grades of methanol, ethanol, N-butanol, Ethyl acetate and other extraction chemical regents used in this study were obtained from Sinopharm Chemical Reagent Co., Ltd. (Shanghai, China). Monodansylcadaverine (MDC) was purchased from Nanjing KeyGen Biotech Co., Ltd. (Nanjing, China). 3-Methyladenine (3-MA) was purchased from Dalian Meilun Biotech Co., Ltd. m-Cherry LC3B plasmid was purchased from Hanheng Biotech Co., Ltd. (Shanghai, China). Lipofectamine 2000 Reagent was purchased from Invitrogen (Grand Island, NY, USA). Primary rabbit polyclonal antibodies against LC3B, p-Akt, Akt, p-P70S6K, p-p38, p38 and JNK were obtained from Cell Signaling Inc. (Danvers, MA, USA). Secondary antibodies, p-ERK, ERK, and GAPDH were obtained from Santa Cruz Biotechnology (Santa Cruz, CA, USA). 2′,7′-dichlorodihydrofluorescein diacetate (DCFH-DA) and CellTiter 96 Aqueous One Solution Cell Proliferation were purchased from Sigma-Aldrich (St. Louis, MO, USA) and Promega (Madison, WI, USA), respectively.

### 4.2. Extraction and Isolation of TFS 

Dried tea flowers (210 g) were pulverized and extracted with 70% methanol two separate times for 2 h under reflux at 60 °C. Seventy per cent methanol extracts were concentrated by a rotary evaporator to obtain a 70% methanol concentrate. The methanol extract was suspended in H_2_O and extracted with equal volumes of ethyl acetate and *N*-butanol three times, successively. The solvents were then evaporated to obtain the ethyl acetate fraction (12.67 g), *N*-butanol fraction (25.50 g) and water fraction (59.71 g). The 25.50 g *N*-butanol fraction was resolved in 10% ethanol and loaded in a D101 macroporous adsorption resin column and successively eluted with 0%, 15%, 30%, 45%, 60%, 75%, and 90% ethanol. The 45%, 60%, 75%, and 90% ethanol eluents were collected, then evaporated and freeze-dried to obtain the 45% ethanol fraction (3.94 g), 60% ethanol fraction (4.17 g), 75% ethanol fraction (3.24 g), and 90% ethanol fraction (0.70 g). The 75% and 90% ethanol fractions were mixed and separated using preparative HPLC to obtain three fractions, TFS fraction 1 (216.8 mg), TFS fraction 2 (281.0 mg), and TFS fraction 3 (126.6 mg). TFS fraction 2 was identified previously and marked as “TFS” in later experiments [17]. 

### 4.3. Cell Lines and Cell Culture

The human ovarian cancer cell line OVCAR-3 was provided by Dr. Jiang from West Virginia University. The cells were cultured in RPMI-1640 medium supplemented with 10% fetal bovine serum (FBS) at 37 °C with 5% CO_2_ in a humidified incubator.

### 4.4. MTS Assay 

OVCAR-3 cells were seeded in a 96-well plate at a density of 1.0 × 10^4^ cells per well and incubated overnight for attachment. The cells were then treated with different concentrations of TFS with or without 3-MA for 24 h. MTS reagents were added to each well and absorbance was measured at 490 nm using a microplate reader. 

### 4.5. Morphology Observation 

OVCAR-3 cells were exposed to different concentrations of TFS (0, 0.5, 1.0, 1.5 μg/mL) for 24 h. Cell morphology was then observed by microscopy (ZEISS, Heidelberg, Germany). Images were captured using a digital camera attached to the microscope at magnification × 10.

### 4.6. Labeling of Autophagic Vacuoles with MDC Staining

OVCAR-3 cells were seeded in a 24-well plate at a density of 1.0 × 10^5^ cells per well after a growth period of 24 h. The cells were then treated with different concentrations of TFS (0, 0.5, 1.0, 1.5 μg/mL) for another 24 h. After media change and wash with PBS, 50 μM MDC was added to each well and the plate was incubated for 30 min, after which fluorescence images were captured using an Olympus DP70 fluorescence microscope.

### 4.7. Transfection of mCherry-LC3B Plasmid

OVCAR-3 cells were grown with medium (without antibiotics) in a 24-well plate (1.0 × 10^5^ cells per well) for 24 h. The cells were ready for transfection once cell confluence reached 90%. The transfection mixture was prepared as follows: 0.5 μg mCherry-LC3B plasmid and 2 μL Lipofectamine 2000 were diluted with 50 μL OPTI-MEMI medium, respectively, and incubated at room temperature for 5 min. The diluted mCherry-LC3B plasmid was mixed with diluted Lipofectamine 2000 to prepare the transfection mixture and incubated at room temperature for 20 min. The culture medium was discarded and washed with PBS twice. Next, 400 μL of serum-free culture medium (without antibiotics) was added to each well and 100 μL of transfection mixture was then added. After a transfection period of 6 h in the incubator, the cells were then incubated with different concentrations of TFS for 24 h. Imaging slides were mounted with DAPI and images were captured using an FV1000 Laser Scanning Confocal Microscope (Olympus, Tokyo, Japan).

### 4.8. Reactive Oxygen Species (ROS) Detection

ROS generation was measured by DCFH-DA staining. Briefly, OVCAR-3 cells were grown in a 96-well plate (1.0 × 10^4^ cells per well) for 24 h and different concentrations of TFS (0, 0.5, 1.0, 1.5 μg/mL) were added to the indicated wells. After a 24 h incubation period, the cells were then incubated with DCFH-DA (10 μM) at 37 °C for 30 min. Imaging slides were mounted with DAPI and images were captured with an Olympus DP70 fluorescence microscope.

### 4.9. Western Blotting

Cells were treated with different concentrations of TFS for 24 h, washed with PBS twice, harvested, and total protein was extracted using protein extraction reagent supplemented with 1% protease inhibitor. Protein content was detected by a BCA protein assay kit. Equal amounts of protein were loaded into 10% SDS-PAGE gels for separation and transferred onto nitrocellulose membranes. The membranes were blocked with 5% non-fat milk for 1 h, then incubated with indicated primary antibody overnight followed by secondary antibody for 2 h at room temperature. Protein bands were visualized using the ChemiDoc MP System (Bio Rad, Hercules, CA, USA). GAPDH was used to normalize relative values of each protein.

### 4.10. Statistical Analysis

Data represents the mean of three independent experiments. Statistical significance was analyzed by Graphpad Prism software using the Newman–Keuls test. * *p* < 0.05, ** *p* < 0.01 and *** *p* < 0.001 were considered as significant *p*-values.

## 5. Conclusions

In the present study, we demonstrated an efficient extraction and purification method for TFS with a yield of 0.34%. We showed for the first time that TFS induced autophagic cell death in ovarian cancer cells. The specific autophagic effect mediated by TFS occurred independently from Akt/mTOR/p70S6K pathway signaling and the TFS-induced autophagy in ovarian cancer cells was found to be accompanied by ERK activation and ROS generation. Thus, our research provides an important basis for future study on the autophagic effects of TFS in ovarian cancer cells.

## Supplementary Materials

The following are available online, Figure S1: Dried tea (*Camellia sinensis*) flowers, Figure S2: Saponins isolated from dried tea (*Camellia sinensis*) flowers, Figure S3: HPLC chromatograms of eluted fractions of 45%, 60%, 75%, 90% ethanol, Figure S4: Chromatograms of TFS 1, TFS 2and TFS 3 detected by LC/MS.

## Abbreviations

TFS	Tea flower saponins
ERK	Extracellular signal-regulated kinase
LC3	Microtubule-associated protein light chain 3
ROS	Reactive oxygen species
MDC	Dansylcadaverine
MAPK	Mitogen-activated protein kinase
p70S6K	p70 ribosomal protein S6 kinase

## References

[1] Piver M.S., Frank T.S., Altchek A., Deligdisch L., Kase N.G. (2003). Epidemiology of Ovarian Cancer. Diagnosis and Management of Ovarian Disorders.

[2] Bast R.C., Hennessy B., Mills G.B. (2009). The biology of ovarian cancer: New opportunities for translation. Nat. Rev. Cancer.

[3] Jayson G.C., Kohn E.C., Kitchener H.C., Ledermann J.A. (2014). Ovarian cancer. Lancet.

[4] Yeung T.-L., Leung C.S., Li F., Wong S.S.T., Mok S.C. (2016). Targeting Stromal-Cancer Cell Crosstalk Networks in Ovarian Cancer Treatment. Biomolecules.

[5] Kim A.D., Kang K.A., Kim H.S., Kim D.H., Choi Y.H., Lee S.J., Kim H.S., Hyun J.W. (2013). A ginseng metabolite, compound K, induces autophagy and apoptosis via generation of reactive oxygen species and activation of JNK in human colon cancer cells. Cell Death Dis..

[6] Huang H., Chen A.Y., Rojanasakul Y., Ye X., Rankin G.O., Chen Y.C. (2015). Dietary compounds galangin and myricetin suppress ovarian cancer cell angiogenesis. J. Funct. Foods.

[7] Gao Y., Rankin G.O., Tu Y.Y., Chen Y.C. (2016). Theaflavin-3, 3′-digallate decreases human ovarian carcinoma OVCAR-3 cell-induced angiogenesis via Akt and Notch-1 pathways, not via MAPK pathways. Int. J. Oncol..

[8] Lee A.H., Su D., Pasalich M., Binns C.W. (2013). Tea consumption reduces ovarian cancer risk. Cancer Epidemiol..

[9] Trudel D., Labbe D.P., Bairati I., Fradet V., Bazinet L., Tetu B. (2012). Green tea for ovarian cancer prevention and treatment: A systematic review of the in vitro, in vivo and epidemiological studies. Gynecol. Oncol..

[10] Rha C.S., Jeong H.W., Park S., Lee S., Jung Y.S., Kim D.O. (2019). Antioxidative, Anti-Inflammatory, and Anticancer Effects of Purified Flavonol Glycosides and Aglycones in Green Tea. Antioxidants.

[11] Naponelli V., Ramazzina I., Lenzi C., Bettuzzi S., Rizzi F. (2017). Green Tea Catechins for Prostate Cancer Prevention: Present Achievements and Future Challenges. Antioxidants.

[12] Way T.-D., Lin H.-Y., Hua K.-T., Lee J.-C., Li W.-H., Lee M.-R., Shuang C.-H., Lin J.-K. (2009). Beneficial effects of different tea flowers against human breast cancer MCF-7 cells. Food Chem..

[13] Wang L., Xu R.J., Hu B., Li W., Sun Y., Tu Y.Y., Zeng X.X. (2010). Analysis of free amino acids in Chinese teas and flower of tea plant by high performance liquid chromatography combined with solid-phase extraction. Food Chem..

[14] Han Q.A., Yu Q.Y., Shi J.A., Xiong C.Y., Ling Z.J., He P.M. (2011). Structural Characterization and Antioxidant Activities of 2 Water-Soluble Polysaccharide Fractions Purified from Tea (*Camellia sinensis*) Flower. J. Food Sci..

[15] Matsuda H., Hamao M., Nakamura S., Kon’i H., Murata M., Yoshikawa M. (2012). Medicinal Flowers. XXXIII. Anti-hyperlipidemic and Anti-hyperglycemic Effects of Chakasaponins I–III and Structure of Chakasaponin IV from Flower Buds of Chinese Tea Plant (*Camellia sinensis*). Chem. Pharm. Bull..

[16] Lin Y.S., Wu S.S., Lin J.K. (2003). Determination of tea polyphenols and caffeine in tea flowers (*Camellia sinensis*) and their hydroxyl radical scavenging and nitric oxide suppressing effects. J. Agr. Food Chem..

[17] Wang Y.M., Ren N., Rankin G.O., Li B., Rojanasakul Y., Tu Y.Y., Chen Y.C. (2017). Anti-proliferative effect and cell cycle arrest induced by saponins extracted from tea (*Camellia sinensis*) flower in human ovarian cancer cells. J. Funct. Foods.

[18] Ouyang L., Shi Z., Zhao S., Wang F.T., Zhou T.T., Liu B., Bao J.K. (2012). Programmed cell death pathways in cancer: A review of apoptosis, autophagy and programmed necrosis. Cell Prolif..

[19] Marino G., Niso-Santano M., Baehrecke E.H., Kroemer G. (2014). Self-consumption: The interplay of autophagy and apoptosis. Nat. Rev. Mol. Cell Biol..

[20] Maiuri M.C., Zalckvar E., Kimchi A., Kroemer G. (2007). Self-eating and self-killing: Crosstalk between autophagy and apoptosis. Nat. Rev. Mol. Cell Biol..

[21] Mrakovcic M., Frohlich L.F. (2018). p53-Mediated Molecular Control of Autophagy in Tumor Cells. Biomolecules.

[22] Li T., Xu X.H., Tang Z.H., Wang Y.F., Leung C.H., Ma D.L., Chen X.P., Wang Y.T., Chen Y., Lu J.J. (2015). Platycodin D induces apoptosis and triggers ERK- and JNK-mediated autophagy in human hepatocellular carcinoma BEL-7402 cells. Acta Pharmacol. Sin..

[23] Sy L.K., Yan S.C., Lok C.N., Man R.Y.K., Che C.M. (2008). Timosaponin A-III Induces Autophagy Preceding Mitochondria-Mediated Apoptosis in HeLa Cancer Cells. Cancer Res..

[24] Chun J., Kang M., Kim Y.S. (2014). A triterpenoid saponin from Adenophora triphylla var. japonica suppresses the growth of human gastric cancer cells via regulation of apoptosis and autophagy. Tumor Biol..

[25] Shen Z.Y., Xu L.Y., Li E.M., Zhuang B.R., Lu X.F., Shen J., Wu X.Y., Li Q.S., Lin Y.J., Chen Y.W. (2008). Autophagy and endocytosis in the amnion. J. Struct. Biol..

[26] Sui Y.X., Yao H., Li S.G., Jin L., Shi P.Y., Li Z.J., Wang G., Lin S.L., Wu Y.J., Li Y.X. (2017). Delicaflavone induces autophagic cell death in lung cancer via Akt/mTOR/p70S6K signaling pathway. J. Mol. Med..

[27] Chang C.H., Lee C.Y., Lu C.C., Tsai F.J., Hsu Y.M., Tsao J.W., Juan Y.N., Chiu H.Y., Yang J.S., Wang C.C. (2017). Resveratrol-induced autophagy and apoptosis in cisplatin-resistant human oral cancer CAR cells: A key role of AMPK and Akt/mTOR signaling. Int. J. Oncol..

[28] Cagnol S., Chambard J.C. (2010). ERK and cell death: Mechanisms of ERK-induced cell death—apoptosis, autophagy and senescence. FEBS J..

[29] Scherz-Shouval R., Shvets E., Fass E., Shorer H., Gil L., Elazar Z. (2007). Reactive oxygen species are essential for autophagy and specifically regulate the activity of Atg4. EMBO J..

[30] Xu X.H., Li T., Fong C.M.V., Chen X.P., Chen X.J., Wang Y.T., Huang M.Q., Lu J.J. (2016). Saponins from Chinese Medicines as Anticancer Agents. Molecules.

[31] Xing J.J., Hou J.G., Liu Y., Zhang R.B., Jiang S., Ren S., Wang Y.P., Shen Q., Li W., Li X.D. (2019). Supplementation of Saponins from Leaves of Panax quinquefolius Mitigates Cisplatin-Evoked Cardiotoxicity via Inhibiting Oxidative Stress-Associated Inflammation and Apoptosis in Mice. Antioxidants.

[32] Shi J.M., Bai L.L., Zhang D.M., Yiu A., Yin Z.Q., Han W.L., Liu J.S., Li Y., Fu D.Y., Ye W.C. (2013). Saxifragifolin D induces the interplay between apoptosis and autophagy in breast cancer cells through ROS-dependent endoplasmic reticulum stress. Biochem. Pharmacol..

[33] Sanchez-Sanchez L., Escobar M.L., Sandoval-Ramirez J., Lopez-Munoz H., Fernandez-Herrera M.A., Hernandez-Vazquez J.M.V., Hilario-Martinez C., Zenteno E. (2015). Apoptotic and autophagic cell death induced by glucolaxogenin in cervical cancer cells. Apoptosis.

[34] Li T., Tang Z.H., Xu W.S., Wu G.S., Wang Y.F., Chang L.L., Zhu H., Chen X.P., Wang Y.T., Chen Y. (2015). Platycodin D triggers autophagy through activation of extracellular signal-regulated kinase in hepatocellular carcinoma HepG2 cells. Eur. J. Pharmacol..

[35] Zhou Y.Y., Li Y., Jiang W.Q., Zhou L.F. (2015). MAPK/JNK signalling: A potential autophagy regulation pathway. Biosci. Rep..

[36] Martinez-Lopez N., Singh R. (2014). ATGs Scaffolds for MAPK/ERK signaling. Autophagy.

[37] Ellington A.A., Berhow M.A., Singletary K.W. (2006). Inhibition of Akt signaling and enhanced ERK1/2 activity are involved in induction of macroautophagy by triterpenoid B-group soyasaponins in colon cancer cells. Carcinogenesis.

[38] Ye Y.C., Wang H.J., Xu L., Liu W.W., Liu B.B., Tashiro S.I., Onodera S., Ikejima T. (2012). Oridonin induces apoptosis and autophagy in murine fibrosarcoma L929 cells partly via NO-ERK-p53 positive-feedback loop signaling pathway. Acta Pharmacol. Sin..

