# Supplementary material for: Saponins Extracted from Tea (Camellia Sinensis) Flowers Induces Autophagy in Ovarian Cancer Cells
Source: Molecules. 2020 Nov 11;25(22):5254. doi: 10.3390/molecules25225254 (PMC7696525; doi:10.3390/molecules25225254)
Supplement: Supplementary file 1 [file molecules-25-05254-s001.pdf]

Saponins extracted from tea (*Camellia sinensis*) flower induces autophagy in ovarian cancer cells

Yaomin Wang[a†], Chen Xia[b†] , Lianfu Chen[a], Yi Charlie Chen[c*], Youying Tu[a**]

[a]Department of Tea Science, Zhejiang University, Hangzhou 310058, P.R. China

[b]Hangzhou Yingshili Biotechnology Co., Ltd, Hangzhou 310000, P.R. China

[c]College of Science, Technology and Mathematics, Alderson Broaddus University, Philippi, WV 26416, USA

[**] Corresponding author. Department of Tea Science, Zhejiang University, 866 Yuhangtang Road, Hangzhou 310058, P.R. China. Tel: +86 0571 88982743; Fax: +86 0571 88982743.

E-mail address: youytu@zju.edu.cn (Y. Tu).

[*] Corresponding author. College of Science, Technology and Mathematics, Alderson Broaddus University, 101 College Hill Drive Philippi, WV 26416, USA. Tel.: +1 304 457 6277; fax: +1 304 457 6239.

E-mail address: chenyc@ab.edu (Y.C. Chen).

† The authors should be considered equal contributors to this paper.

Supplementary Figures

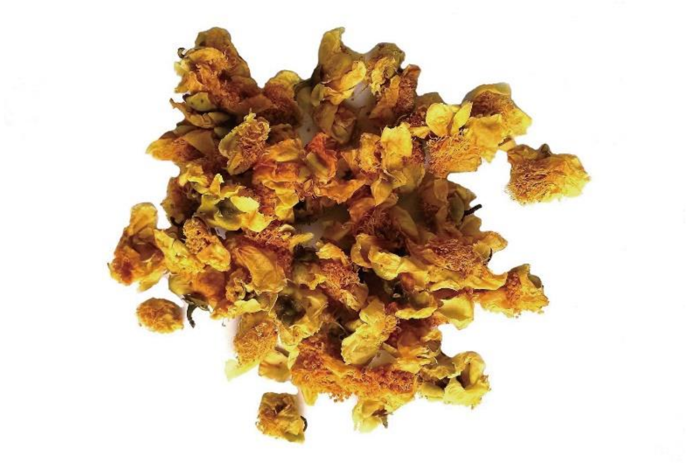

Figure S1. Dried tea (Camellia sinensis) flowers.

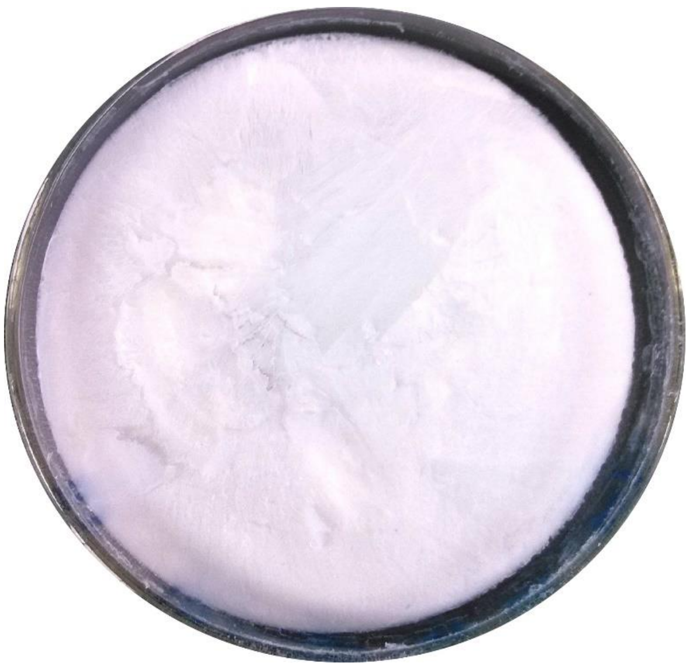

Figure S2. Saponins isolated from dried tea (Camellia sinensis) flowers.

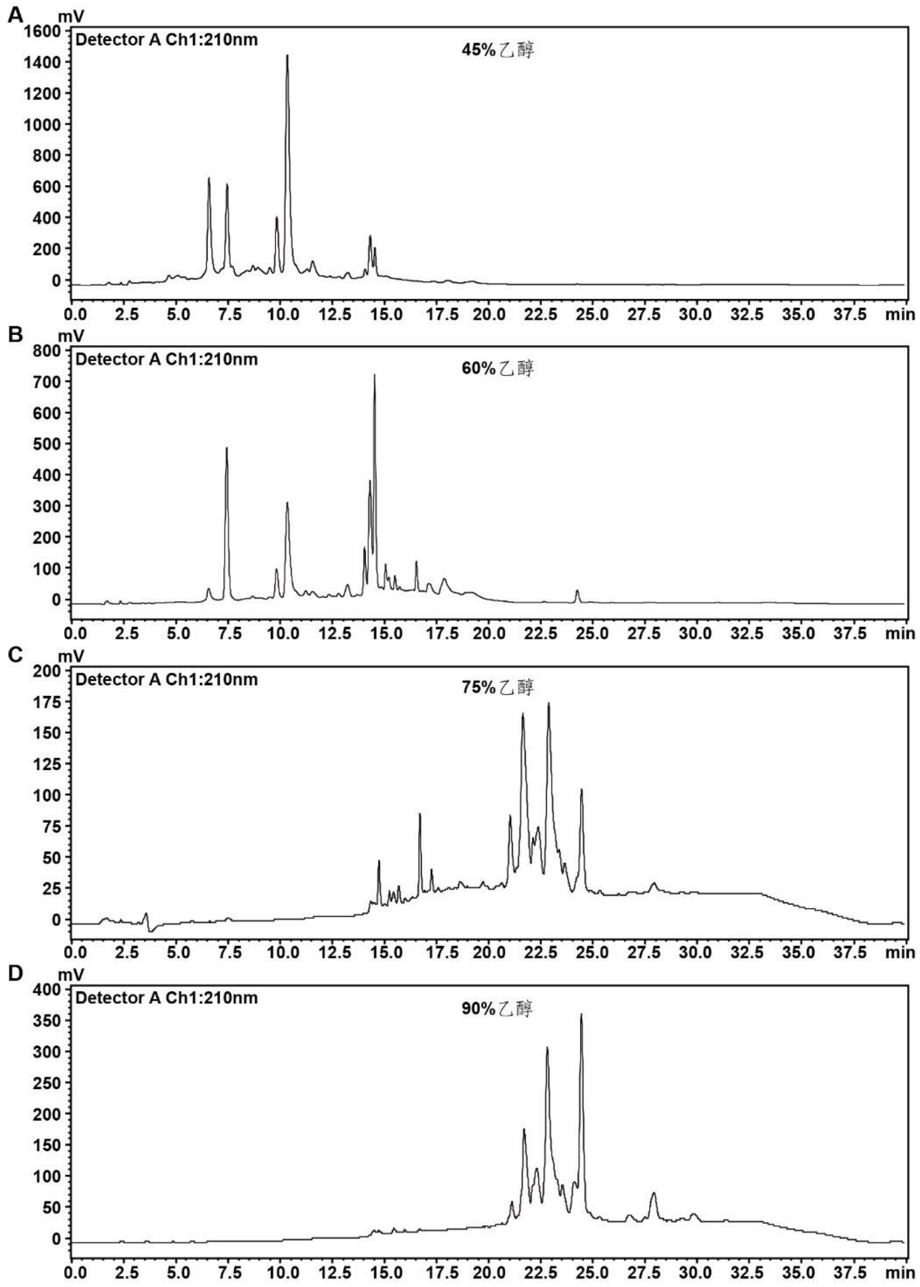

Figure S3. HPLC chromatograms of eluted fractions of 45%, 60%, 75%, 90% ethanol.

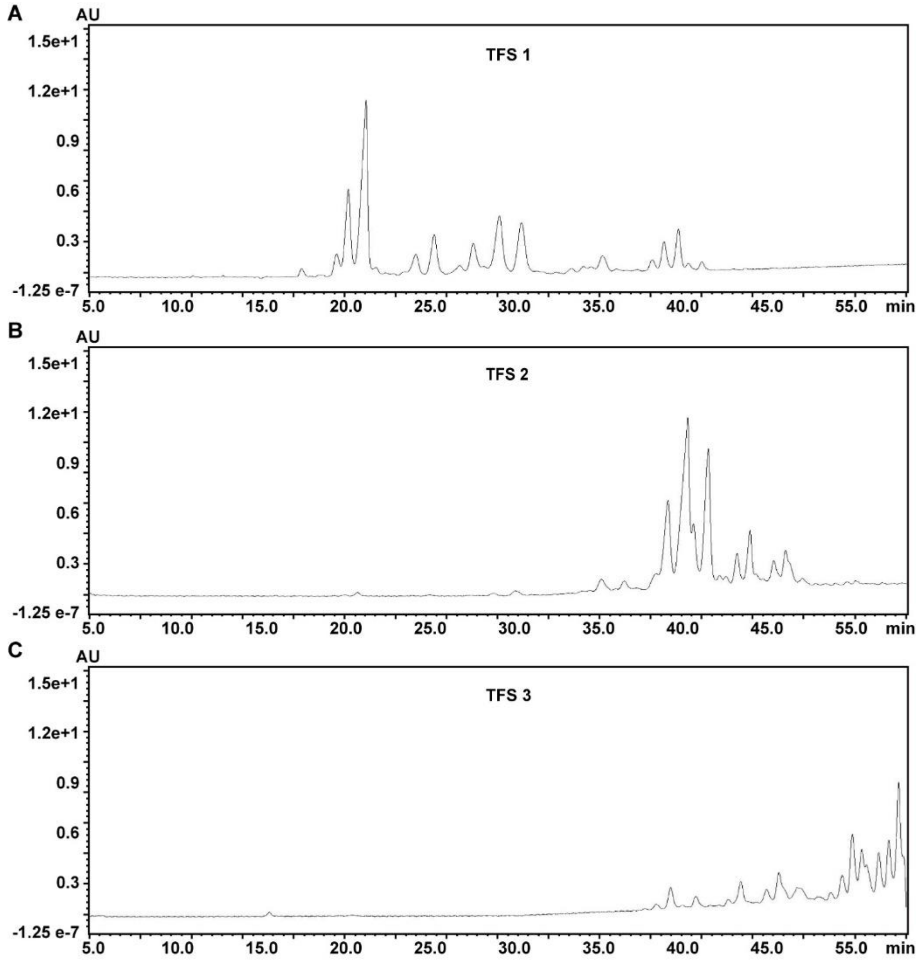

Figure S4. Chromatograms of TFS 1, TFS 2and TFS 3 detected by LC/MS.
